# The Impact of Obesity on Left Ventricular Assist Device Outcomes

**DOI:** 10.3390/medicina56110556

**Published:** 2020-10-23

**Authors:** Konstantin Zhigalov, Michel Pompeu Barros Oliveira Sá, Arian Arjomandi Rad, Robert Vardanyan, Lukas Goerdt, Thomas Chrosch, Alina Zubarevich, Daniel Wendt, Nikolaus Pizanis, Achim Koch, Markus Kamler, Rafal Berger, Bastian Schmack, Arjang Ruhparwar, Aron-Frederik Popov, Alexander Weymann

**Affiliations:** 1 Department of Thoracic and Cardiovascular Surgery, West German Heart and Vascular Center Essen, University Hospital of Essen, University Duisburg-Essen, 45122 Essen, Germany; konstantin.zhigalov@yahoo.com (K.Z.); lukasgoerdt@yahoo.de (L.G.); thomas.chrosch@unitybox.de (T.C.); alina.zubarevich@gmail.com (A.Z.); daniel.wendt@uk-essen.de (D.W.); Nikolaus.Pizanis@uk-essen.de (N.P.); Achim.Koch@uk-essen.de (A.K.); Bastian.Schmack@uk-essen.de (B.S.); Arjang.Ruhparwar@uk-essen.de (A.R.); alexander.weymann@uk-essen.de (A.W.); 2 International Thoracic and Cardiovascular Research Association (ITCVR), 26133 Oldenburg, Germany; michel_pompeu@yahoo.com.br (M.P.B.O.S.); robert.vardanyan16@imperial.ac.uk (R.V.); aronf.popov@gmail.com (A.-F.P.); 3 Department of Cardiovascular Surgery at the Pronto Socorro Cardiológico de Pernambuco (PROCAPE), Recife, University of Pernambuco, Recife 74970-240, Brazil; 4 Department of Medicine, Faculty of Medicine, Imperial College London, London SW7 2AZ, UK; 5 Department of Cardiothoracic Surgery, Heart Center Essen Huttrop, University Hospital Essen, 45138 Essen, Germany; markus.kamler@heh.uk-essen.de; 6 Department of Thoracic and Cardiovascular Surgery, University Medical Centre Tubingen, 72076 Tubingen, Germany; Rafal.Berger@med.uni-tuebingen.de

**Keywords:** left ventricular assist device, obesity, mechanical circulatory support, heart failure, cardiac assist and artificial heart

## Abstract

*Background and Objectives*: The understanding of high body mass index (BMI) and outcomes after Left Ventricular Assist Device (LVAD) implantation continues to evolve and the relationship has not been established yet. In this study, we investigated the effects of obesity (BMI > 30 kg/m^2^) on post–LVAD implantation outcomes. HeartWare LVAD and Heart Mate III LVAD were implanted. The primary outcome that was measured was mortality (in-hospital and on follow-up). The secondary outcomes that were measured were major adverse events. *Materials and Methods***:** At our institution, the West German Heart and Vascular Center (Essen, Germany), from August 2010 to January 2020, a total of 210 patients received a long-term LVAD. Patients were stratified according to BMI ≥ 30 kg/m^2^ representing the obesity threshold. The first group (*n* = 162) had an average BMI of 24.2 kg/m^2^ (±2.9), and the second group (*n* = 48) had an average BMI of 33.9 kg/m^2^ (±3.2). Baseline demographics were analysed alongside comorbidities per group. *Results:* Overall mortality was not significantly different between the obese group (51.1% *n* = 24) and the nonobese group (55.2%, *n* = 85) (*p* = 0.619). The difference between the mean duration of survival of patients who expired after hospital discharge was insignificant (2.1 years ± 1.6, group 1; 2.6 years ± 1.5, group 2; *p* = 0.29). In-hospital mortality was unvaried between the two groups: group 1: *n* = 34 (44% out of overall group 1 deaths); group 2: *n* = 11 (45.8% out of overall group 2 deaths) (*p* > 0.05). Postoperative complications were unvaried between the obese and the non-obese group (all with *p* > 0.05). However, a significant difference was found with regards to follow-up neurological complications (18.5% vs. 37.8%, *p* = 0.01) and LVAD thrombosis (14.7% vs. 33.3%, *p* = 0.01), as both were higher in the obese population. *Conclusion:* Obesity does not form a barrier for LVAD implantation in terms of mortality (in-hospital and on follow up). However, a significantly higher incidence of follow-up LVAD thrombosis and neurological complications has been found in the obese group of patients.

## 1. Introduction

Obesity is widely recognized as a major risk factor for adverse cardiovascular outcomes, and chronic conditions such as diabetes, hypertension, and heart failure [1]. It has been also linked to increased mortality and morbidity in patients with cardiovascular diseases [2]. Even in the absence of traditional risk factors for heart failure, chronic obesity has been linked to structural myocardial changes and increasing diastolic and systolic dysfunction [3].

The growing worldwide prevalence of obesity (defined as body mass index (BMI) > 30 kg/m^2^) and its associated comorbidities has been putting increased pressure on healthcare systems, affecting 10–30% of adults in Europe and over one third of the adult population in the US [4,5]. The following has also been linked to a consequent rise in heart failure prevalence, thus increasing the need for treatment.

Heart Transplantation (HT) has been considered the gold standard with regards to treating patients with end-stage cardiomyopathy [6]. Nevertheless, a vast plethora of evidence has shown that obesity might be a contraindication for HT. Indeed, obese patients have poorer post transplantation survival, increased long term complication, and worse cardiovascular outcomes.

Despite obesity being a contraindication to HT, the evidence around the outcomes of left ventricular assist devices (LVADs) in obesity has been scarce and contradicting [7]. Thanks to years of advancements in technology, LVADs are serving an increasing number of patients as both a bridge to transplantation (BTT) or as a destination therapy (DT) [8,9,10].

The present study sought to contribute to the current body of evidence regarding LVAD implantation in obese patients and the related post-LVAD implantation outcomes. The primary outcome was mortality (in-hospital and on follow-up) while the secondary outcomes were adverse events following LVAD implantation.

## 2. Methods

### 2.1. Study Population

At our institution, the West German Heart and Vascular Center (Essen, Germany), from August 2010 to January 2020, a total of 210 patients received a long-term LVAD because of HF by cardiomyopathy, post-cardiotomy shock (PCS), or acute myocardial infarction as DT, BTT, and bridge to candidacy. The indication for the operation was made following the current guidelines. We used one of two LVAD models: HeartMate III (HM III) (Thoratec Corp., Pleasanton, CA, USA) and HeartWare (HVAD) (HeartWare International Inc., Framingham, MA, USA). Choice of VAD was based on the availability of the device in the clinic and the personal decision of the surgeon. All our patients underwent surgery through median sternotomy.

### 2.2. Study Design

The study is a retrospective review of prospectively collected data. Data collected as part of the institutional Mechanical Circulatory Support Database included detailed information on patients’ demographics, baseline clinical characteristics, and their laboratory, echocardiographic and hemodynamic parameters, as well as intraoperative variables and postoperative outcomes. The follow-up was carried out for all patients in June 2020 in our VAD clinic. The follow-up period was 2.4 years (±2.34). The study was approved by the local ethics committee.

### 2.3. Study Groups

Patients were stratified according to BMI ≥ 30 kg/m^2^ representing the obesity threshold. Group 1 (*n* = 162) had an average BMI of 24.2 kg/m^2^ (±2.9) and represented the LVAD non-obese group, and the second group (*n* = 48) had an average BMI of 33.9 kg/m^2^ (±3.2) and represented the LVAD obese group.

### 2.4. Outcome Measures

The primary endpoint was mortality (in-hospital and on follow-up) while the secondary endpoints were adverse events and other postoperative characteristics following LVAD implantation. Patients were censored after their death or at the cutoff of the study.

### 2.5. Variables and Definitions

Variables were evaluated, including baseline characteristics, as well as further preoperative clinical data, preoperative laboratory parameters, intraoperative data, postoperative variables, and follow-up data. The adverse events were defined according to “INTERMACS Adverse Event Definitions” [11].

### 2.6. Anticoagulation Regimen

Our anti-coagulation regimen follows the previously described one by Pilarczyk et al. (2019) [12] and included intravenous unfractionated Heparin once chest tube drainage was <50 mL/h to maintain an activated partial thromboplastin time of 50 to 60 s. Acetylsalicylic acid (100–300 mg/day) was started 48–72 h after LVAD implantation. Once the patient was stable (chest tubes removed and return of GI function), Heparin was replaced by Warfarin. Warfarin was adjusted to achieve an international normalized ratio (INR) of 2.2–2.8. Continuous venovenous hemofiltration was used to treat all of our patients. To prevent clot formation it the circuit, regional anticoagulation was achieved though citrate, and systemic anticoagulation was achieved through intravenous heparin.

### 2.7. Statistical Analysis 

The data were analyzed using IBM SPSS version 25 (IBM Corp., Chicago, IL, USA). We used the Kolmogorov–Smirnov test to prove the data for normal distribution. Quantitative data are expressed as the mean and standard deviation (SD) for normally distributed variables and as the median and interquartile range for not normally distributed variables. Categorical data are expressed as frequency and percentage. We used the Mann–Whitney U test to compare mean values and the chi-square test to examine the distribution of categorical variables between the groups. We used the Kaplan–Meier method to analyze the survival. The significance of survival differences between the groups was assessed with Log-Rank and Breslow tests. A value of *p* < 0.05 was considered to be statistically significant.

## 3. Results

### 3.1. Baseline Characteristics

A total of 210 patients implanted with Heartmate III (HMIII) and Heartware (HVAD) LVADs from 2010 to 2020 were enrolled in this study. In the total population, 43 patients (35%) were implanted as BTT and 155 (74%) as DT. The obese cohort (group 2) consisted of 48 patients (23%) with BMI > 30 kg/m^2^, and the nonobese group (group 1) comprised of 162 nonobese patients (77%) with BMI < 30 kg/m^2^. The mean BMI in the obese group was 33.9 kg/m^2^ compared with 24.2 kg/m^2^ in the in the non-obese group (*p* < 001; Table 1). Group 1 consisted of 21.6% females with an average age of 57.7 years (±12.1) while group 2 consisted of 8.3% females (*p* < 0.05) with an average age of 56.8 years (±3.3) (*p* > 0.05).

Baseline tabulation of comorbidities (Table 1) revealed no major statistical difference between the two groups except for increased incidence in the obese group of hypertension (82.3% vs. 63.6%; *p* < 0.05) and of Coronary Artery Disease (CAD) (70.8% vs. 54.7%; *p* < 0.05). The groups 1 and 2 did not show any other significant difference in baseline preoperative characteristics, cardiorespiratory conditions, device strategy, and laboratory parameters (Table 1 and Table 2).

### 3.2. Intraoperative Characteristics 

There were no significant differences in procedure durations, distribution of LVAD models, and concomitant procedures between the groups (Table 3). All of our patients (100%) underwent surgery through median sternotomy.

### 3.3. Survival Data and Adverse Events

The follow-up period was 2.4 years (±2.34). Table 4 outlines survival data. Overall mortality associated with LVAD implantation was found not to be significantly different between the obese patients group (51.1% *n* = 24) and the nonobese group (55.2%, *n* = 85) (*p* = 0.619). The difference between the mean duration of survival of patients who expired after hospital discharge was insignificant (2.1 years ±1.6, group 1; 2.6 years ± 1.5, group 2; *p* = 0.29). In-hospital mortality was unvaried between the two groups. Group 1: *n* = 34 (44% out of overall group 1 deaths); group 2: *n* = 11 (45.8% out of overall group 2 deaths).

All death causes of mortality identified were insignificant between group 1 and 2: cardiopulmonary failure (18.8% ± 0.39 vs. 33.3% ± 0.48%; *p* = 0.13), multiorgan failure (48.2% ± 0.5 vs. 33.3% ± 0.48; *p* = 0.19), bleeding (4.7% ± 0.21 vs. 4.17% ± 0.2%; *p*= 0.91), infection (27.1% ± 0.45 vs. 25% ± 0.44; *p* = 0.84), cerebrovascular accident (21.2% ± 0.4 vs. 33.3% ± 0.48; *p* = 0.22), and unknown (15.3% ± 0.36 vs. 16.7% ± 0.38; *p* = 0.87).

Postoperative complications (Table 5) were unvaried between the obese and the non-obese group: stroke (4.3% vs. 6.3%; *p* = 0.60), intracranial bleeding (4.4% vs. 3.4%; *p* = 0.14), hypoxic encephalopathy (3.2% vs. 2.3% *p*= 0.22), and neurological complications (10.5% vs. 9.0%; *p* = 0.18). The difference in postoperative reoperation due to bleeding was also not significant (20.9% vs. 21.9%; *p* = 0.5). Nevertheless, the postoperative rate of pneumonia was found to be significantly higher in the non-obese population (25% vs. 8.1%; *p* = 0.03).

One-hundred fifty-two patients (72.4%) survived at follow up, 116 in group 1 and 36 in group 2. On follow-up (Table 6) no significant differences were found in terms of stroke (10.3% vs. 16.7%; *p* = 0.31), GI bleeding (20.6% vs. 13.9%; *p* = 0.36), device malfunction (7.0% vs. 2.8%; *p* = 0.36), follow up right heart failure (6.6% vs. 7.0%; *p* = 0.78), hypoxic encephalopathy (2.0% vs. 0.3%; *p* = 0.70), intracranial bleeding (13.8% vs. 19.4%; *p* = 0.41), and driveline infection (39.5% vs. 37.1%; *p* = 0.28). However, a significant difference between group 1 and 2 was found with regards to follow up neurological complications (18.5% vs. 37.8%; *p* = 0.01) and LVAD thrombosis (14.7% vs. 33.3%; *p* = 0.01).

## 4. Discussion

Obesity has been extensively described in the published literature as an independent risk factor for heart failure and a contraindication for heart transplant [13]. Thus, obesity limits the treatment choice available for this vulnerable and high-risk category of patients in the case of end stage cardiomyopathy. Despite the advances made in the past decades with regards to LVAD technology and its related outcomes, the evidence in obesity remains scarce with various contradicting findings. Traditionally, obese patients have been thought and assumed to be more vulnerable to LVAD implantation due to their increased risks of right ventricular failure, respiratory failure, and infections rate [14,15,16]. Obesity has also been found to be an independent predictor of post cardiac surgery infections, thus further exposing this category of patients to increased risks [17,18]. However, LVAD could be considered as the only pathway to heart transplantation, especially when taking into consideration the high mortality rates of bariatric surgery [19]. In a small-scale clinical trial, Dhesi et al. (2011) evaluated the role of LVAD as a “bridge to weight loss” prior to heart transplantation [20]. Despite their limited cohort of 19 patients, for the first time, their research has shown that LVAD could be successfully used as a bridge to weight loss in patients with heart failure, potentially considering them for heart transplantation candidacy. Their results have also been supported by Clerkin et al. (2016), who among 3586 patients evaluated the impact of obesity on BT LVAD patients and found patients with obesity to have similar freedom from death or delisting to non-obese patients, and while weight-loss was uncommon, it was possible [21]. Nevertheless, their results did also show that Class II and greater obese patients presented with greater rates of complications.

The following study assessed the in-hospital and the follow up mortality of 210 patients who underwent LVAD implantation at our center, with 162 patients in the non-obese LVAD group and 48 patients in the obese LVAD group. Our study found no significant difference in both in hospital mortality and follow up mortality (average follow-up period of 2.2 years) between the obese LVAD group and non-obese LVAD group. The findings of the following analysis are supported by those of numerous other reports that found no difference in in-hospital mortality, two years and three years follow up mortality between obese, and non-obese patients undergoing LVAD implantation [13,21,22,23]. A large study by Brewer et al. that reported on 896 Heart Mate II (HMII) patients has shown no differences in two-year survival between obese and non-obese patients, but increased rehospitalization rates in the obese group [24]. A further study by Mohamedali et al. conducted on 288 HMII and HVAD patients found that despite higher readmission rates, obesity was not associated with a decrease in survival rates at three years post LVAD implantation [25]. Greater insight was provided by Yost et al., who in their analysis also included a group of extremely obese (BMI > 35 kg/m^2^) patients receiving HMII and HVAD LVADs [26]. On assessment of 30-day, 1-, and 2- years follow neither the obese nor the extremely obese showed any significant difference in survival (Table 4). Postoperative adverse events and complications including length of stay, sternal infection, driveline/pocket infection, systemic infection, GI-bleeding, and neurological events were also found to be unvaried across groups in the study [26]. Nevertheless, studies in the past have found severely obese (BMI ≥ 35 kg/m^2^) patients to have worse outcomes on LVAD. Musci et al. (2008) found that severe obesity carries a significant risk with an almost six-fold risk of the combined endpoint of postoperative mortality and failure of procedural success [27].

While LVAD’s have contributed to significantly improving the survival of patients with stage D heart failure in the general population, complications are among their weaknesses [28]. Firstly, the results of our analysis found obesity to be associated with increased risks of LVAD thrombosis (14.7% vs. 33.3%; *p* = 0.01). Obesity is known to promote chronic inflammation and impaired fibrinolysis, thus exposing patients to higher risks of thrombosis [29]. Furthermore, obesity could lead to higher risk of thrombosis through the action of adipocytokines, such as leptin, adiponectin, and Resistin [30]. Supporting evidence demonstrating the relationship between LVAD thrombosis and obesity has been widely published in the literature. Han et al. studied 164 patients with the HMII LVAD implant and found LVAD thrombosis to be significantly higher in patients with obesity while reporting no decrease in the two-year survival among the obese group [31]. Secondly, the following study found a significant difference with regards to neurological complications (18.5% vs. 37.8%; *p* = 0.01). While knowledge about the impact of neurological complications on LVAD outcomes is scarce, current research has related them to poorer outcomes. In an analysis of 17,735 LVAD patients, a prevalence of 7.6% of neurological complications was found, which correlated with longer hospital stays and higher mortality rates [32].

This study does not demonstrate an increase in in-hospital and follow up mortality (2.2 years) following LVAD implantation in patients with obesity compared to non-obese individuals. However, a significantly higher incidence of LVAD thrombosis and neurological complications has been found in the obese group of patients.

## 5. Study Limitations

This study is a retrospective non-randomized analysis of a relatively small number of CF-LVAD patients from a single medical center over a span of 10 years. Clinical decisions were made in a non-blinded fashion. Differences in outcomes between HeartMate III and HeartWare should also be explored in future studies.

## 6. Conclusions

Obesity does not form a barrier for LVAD implantation in terms of mortality (in-hospital and on follow up). Furthermore, postoperative and follow-up major adverse events did not significantly differ for the obese and non-obese populations receiving LVAD. However, a significantly higher incidence of LVAD thrombosis and neurological complications has been found in the obese group of patients. Although greater scale multicenter clinical trials are needed, LVAD could offer great benefits to obese patients with heart failure who are ineligible for heart transplantation, with its use also being possibly considered as the only bridge to transplantation.

## Figures and Tables

**Table 1:** Baseline characteristics of the study patients.

Characteristics	Total	Group 1 (Non-Obese)	Group 2 (Obese)	*p*-Value
Demographic data				
Number of patients, *n*	210	162	48	-
Age, years	57.5 (±11.5)	57.7 (±12.1)	56.8 (±9.3)	-
Female, *n* (%)	39 (18.6%)	35 (21.6%)	4 (8.3%)	**0.03**
Body mass index, kg/m^2^	26.4 (±5.1)	24.2 (±2.9)	33.9 (±3.2)	**<0.01**
BSA	2.0 (±0.2)	1.9 (±0.2)	2.3 (±0.2)	**<0.01**
Comorbidities, *n* (%)				
Arterial hypertension	142 (67.6%)	103 (63.6%)	39 (81.25%)	**0.02**
Coronary artery disease	122 (58.4%)	88 (54.7%)	34 (70.8%)	**0.04**
Hyperlipidaemia	102 (48.6%)	76 (46.9%)	26 (54.2%)	0.38
Smoking history	122 (58.4%)	90 (55.9%)	32 (66.7%)	0.18
Atrial fibrillation	86 (41.5%)	61 (38.1%)	25 (53.2%)	0.07
Diabetes	71 (34.0%)	50 (31.1%)	21 (43.75%)	0.10
Disease of peripheral arteries	29 (13.9%)	25 (15.5%)	4 (8.3%)	0.21
Chronic obstructive pulmonary disease	45 (21.5%)	33 (20.5%)	12 (25%)	0.51
Stroke	13 (6.2%)	12 (7.5%)	1 (2.1%)	0.18
Primary diagnosis, *n* (%)				
Ischemic cardiomyopathy	101 (48.1%)	76 (46.9%)	25 (52.1%)	0.53
Dilated cardiomyopathy	101 (48.1%)	79 (48.7%)	22 (45.8%)	0.72
Toxic cardiomyopathy	4 (1.9%)	4 (2.5%)	0	0.27
Other cardiomyopathy	4 (1.9%)	3 (1.9%)	1 (2.1%)	0.92
Acute myocardial infarction	92 (44.4%)	70 (43.75%)	22 (46.8%)	0.71
Cardiorespiratory conditions				
Mechanical ventilation, *n* (%)	44 (21.3%)	35 (21.9%)	9 (19.1%)	0.69
Ejection fraction, %	17% (±6.9)	16.5% (±7.0)	18.8% (±6.0)	0.05
INTERMACS profile, *n* (%)				
1–3	158 (75.2%)	124 (76.5%)	34 (70.8%)	0.80
4–7	52 (24.8%)	38 (23.5%)	14 (29.2%)	0.29
Device strategy at the time of implantation, *n* (%)				
Destination therapy	155 (73.8%)	118 (72.8%)	37 (77.1%)	0.56
Bridge to candidacy	12 (9.7%)	10 (9.8%)	2 (9.1%)	0.50
Bridge to transplant	43 (34.7%)	34 (33.3%)	9 (40.9%)	0.92

The bold values are those with the *p* < 0.05. BSA: body surface area; INTERMACS: Interagency Registry for Mechanically Assisted Circulatory Support.

**Table 2:** Preoperative laboratory parameters.

Characteristics	Total	Group 1 (Non-Obese)	Group 2 (Obese)	*p*-Value
WBC, × 10^9^/L	9.5 (±3.8)	9.4 (±3.8)	9.7 (±3.5)	0.68
CRP, mg/L	4.6 (±5.2)	4.5 (±5.1)	5.2 (±5.9)	0.50
Creatinine, mg/dL	1.5 (±0.9)	1.5 (±0.7)	1.6 (±1.4)	0.42
Total bilirubin, mg/dL	1.4 (±1.3)	1.3 (±1.3)	1.5 (±1.6)	0.50
ALT, U/L	110.7 (±293.0)	110.5 (±314.5)	111.3 (±208.1)	0.98
LDH, U/L	410.1 (±516.2)	416.8 (±568.2)	387.9 (±287.6)	0.69

WBC: white blood cell; BUN: blood urea nitrogen; ALT: alanine aminotransferase; CRP: C-reactive protein; LDH: lactate dehydrogenase.

**Table 3:** Intraoperative data.

Characteristics	Total	Group 1 (Non-Obese)	Group 2 (Obese)	*p*-Value
Durations, min				
Operation	218.6 (±72.6)	215.7 (±70.6)	228.3 (±79.2)	0.32
Cardiopulmonary bypass	90.2 (±34.8)	88.7 (±32.2)	95.2 (±42.5)	0.37
LVAD model, *n* (%)				
HeartMate III	22 (10.5%)	17 (10.5%)	5 (10.4%)	0.99
HeartWare	188 (89.5%)	145 (89.5%)	43 (89.6%)	0.99
Isolated procedure, *n* (%)	182 (86.7%)	142 (87.7%)	40 (83.3%)	0.44
Combined procedure, *n* (%)	28 (13.3%)	20 (12.3%)	8 (16.7%)	0.44
Concomitant procedures (also in various combinations), *n* (%)				
Tricuspid valve surgery	7 (3.4%)	4 (2.6%)	3 (6.4%)	0.21
Atrium septum defect closure	3 (1.5%)	2 (1.3%)	1 (2.1%)	0.67
Aortic valve replacement	11 (5.4%)	9 (5.8%)	2 (4.3%)	0.69
Left ventricular aneurysm	3 (1.5%)	3 (1.9%)	0	0.34
Coronary artery bypass graft	3 (1.5%)	2 (1.3%)	1 (2.1%)	0.67
Ventricular septum defect closure	3 (1.5%)	3 (1.9%)	0	0.34

LVAD: left ventricular assist device.

**Table 4:** Survival data.

Characteristics	Total	Group 1 (Non-Obese)	Group 2 (Obese)	*p*-Value
Survival, %				
30-day survival	177/210 (84.2%)	135/162 (83.3%)	42/48 (87.5%)	0.42
1-year survival	128/199 (64.3%)	97/154 (63.0%)	31/45 (68.9%)	0.47
2-year survival	106/183 (57.9%)	79/141 (56.0%)	27/42 (64.3%)	0.34
Causes of death, *n* (%)				
Right heart failure	24 (11.9%)	16 (10.4%)	8 (17.0%)	0.22
Infection	29 (14.4%)	23 (14.9%)	6 (12.8%)	0.71
Cerebrovascular accident	26 (12.9%)	18 (11.7%)	8 (17.0%)	0.34
Multiorgan failure	49 (24.4%)	41 (26.6%)	8 (17.0%)	0.18
Bleeding	5 (2.5%)	4 (2.6%)	1 (2.1%)	0.86
Unknown	17 (8.5%)	13 (8.4%)	4 (8.5%)	0.99

**Table 5:** Major postoperative adverse events.

Characteristics	Total	Group 1 (Non-Obese)	Group 2 (Obese)	*p*-Value
**Need for revision due to bleeding**	45 (22.0%)	33 (20.9%)	12 (25.5%)	0.50
**Thromboembolism, *n* (5)**	2 (1.0%)	1 (0.6%)	1 (2.1%)	0.36
**Major infection, *n* (%)**	67 (31.9%)	56 (34.6%)	11 (23.0%)	0.13
Driveline infection	5 (2.4%)	3 (1.9%)	2 (4.3%)	0.36
Pneumonia	34 (21.1%)	31 (25%)	3 (8.1%)	0.03
Sepsis	35 (17.0%)	31 (19.5%)	4 (8.5%)	0.08
**Respiratory failure, *n* (%)**	87 (41.4%)	70 (43.2%)	17 (35.4%)	0.34
Ventilation over 6 days post-implant	83 (40.5%)	66 (41.8%)	17 (36.2%)	0.49
Reintubation	39 (19.0%)	32 (20.3%)	7 (14.9%)	0.41
Tracheostomy	52 (25.3%)	41 (25.9%)	11 (23.4%)	0.72
**Right heart failure, *n* (%)**	105 (54.1%)	80 (53.7%)	25 (55.6%)	0.72
Need for inotropes over 14 days postimplant	63 (32.5%)	50 (33.6%)	13 (28.9%)	0.56
ST-RVAD, intraoperative implantation	10 (4.9%)	7 (4.5%)	3 (6.4%)	0.60
ST-RVAD, postoperative implantation	11 (5.4%)	8 (5.1%)	3 (6.4%)	0.72
**Hepatic dysfunction, *n* (%)**	20 (9.8%)	17 (10.8%)	3 (6.4%)	0.37
**Acute renal dysfunction, *n* (%)**	92 (44.9%)	71 (44.9%)	21 (44.7%)	0.98
**Neurological dysfunction, *n* (%)**	19 (9.0%)	17 (10.5%)	2 (4.2%)	0.18
Ischemic stroke	12 (5.6%)	10 (6.3%)	2 (4.3%)	0.60
Intracranial haemorrhage	7 (3.4%)	7 (4.4%)	0	0.14
Hypoxic encephalopathy	5 (2.3%)	5 (3.2%)	0	0.22
**Psychiatric episode, *n* (%)**	6 (2.9%)	5 (3.2%)	1 (2.1%)	0.71

**Table 6:** Major follow-up adverse events.

Characteristics	Total (*n* = 152)	Group 1 (Non-Obese *n* = 116)	Group 2 (Obese *n* = 36)	*p*-Value
Mean number of readmissions per patients (±standard deviation)	3.7 (±4.3)	3.7 (±4.6)	3.6 (±3.3)	0.89
Stroke	18 (11.8%)	12 (10.3%)	6 (16.7%)	0.31
Intracranial bleeding	23 (15.1%)	16 (13.8%)	7 (19.4%)	0.41
Hypoxic encephalopathy	3 (2.0%)	2 (1.7%)	1 (0.3%)	0.70
Neurological complications	37 (23%)	23 (18.5%)	14 (37.8%)	**0.01**
Thoracic bleeding	13 (40.5%)	10 (8.6%)	3 (8.3%)	0.96
GI bleeding	29 (19.1%)	24 (20.6%)	5 (13.9%)	0.36
LVAD Thrombosis	29 (19.1%)	17 (14.7%)	12 (33.3%)	**0.01**
Driveline infection	60 (39.5%)	43 (37.1%)	17 (47.2%)	0.28
Device malfunction	9 (5.9%)	8 (7.0%)	1 (2.8%)	0.36
Right heart failure	10 (6.6%)	8 (7.0%)	2 (5.5%)	0.78

Bold: significant difference (*p* < 0.05).

## References

[1] Pi-Sunyer X. (2009). The medical risks of obesity. Postgrad. Med..

[2] Doehner W., Erdmann E., Cairns R., Clark A.L., Dormandy J.A., Ferrannini E., Anker S.D. (2012). Inverse relation of body weight and weight change with mortality and morbidity in patients with type 2 diabetes and cardiovascular co-morbidity: An analysis of the PROactive study population. Int. J. Cardiol..

[3] Ebong I.A., Goff D.C., Rodriguez C.J., Chen H., Bertoni A.G. (2014). Mechanisms of heart failure in obesity. Obes. Res. Clin. Pract..

[4] Marques A., Peralta M., Naia A., Loureiro N., de Matos M.G. (2018). Prevalence of adult overweight and obesity in 20 European countries, 2014. Eur. J. Public Health.

[5] Ogden C.L. (2007). Obesity among Adults in the United States—No Change Since 2003–2004.

[6] Alraies M.C., Eckman P. (2014). Adult heart transplant: Indications and outcomes. J. Thorac. Dis..

[7] Mehra M.R., Kobashigawa J., Starling R., Russell S., Uber P.A., Parameshwar J., Mohacsi P., Augustine S., Aaronson K., Barr M. (2006). Listing criteria for heart transplantation: International Society for Heart and Lung Transplantation guidelines for the care of cardiac transplant candidates—2006. J. Heart Lung Transplant..

[8] Zhigalov K., Mashhour A., Szczechowicz M., Mkalaluh S., Karagezian S., Gogia I., Isaev M., Kadyraliev B.K., Easo J., Ennker J. (2018). Clinical Outcome and Comparison of three different left ventricular assist devices in a high-risk cohort. Artif. Organs.

[9] Zhigalov K., Szczechowicz M., Mashhour A., Mkalaluh S., Safonov D., Enginoev S., Easo J., Ennker J., Eichstaedt H.C., Weymann A. (2019). Impact of preoperative extracorporeal life support on left ventricular assist device outcomes: A comparative study. Int. J. Artif. Organs.

[10] Zhigalov K., Mashhour A., Szczechowicz M., Mkalaluh S., Baranov A., Easo J., Altmann S., Eichstaedt H.C., Weymann A. (2019). Long-Term Left Ventricular Assist Device (LVAD): A Rare Case of 10 Years’ Support and Follow-Up. Am. J. Case Rep..

[11] Stevenson L.W., Pagani F.D., Young J.B., Jessup M., Miller L., Kormos R.L., Naftel D.C., Ulisney K., Desvigne-Nickens P., Kirklin J.K. (2009). INTERMACS profiles of advanced heart failure: The current picture. J. Heart Lung Transplant..

[12] Pilarczyk K., Carstens H., Papathanasiou M., Luedike P., Koch A., Jakob H., Kamler M., Pizanis N. (2020). Prediction of acute kidney injury after left ventricular assist device implantation: Evaluation of clinical risk scores. Artif. Organs.

[13] Baena-Díez J.M., Byram A.O., Grau M., Gómez-Fernández C., Vidal-Solsona M., Ledesma-Ulloa G., González-Casafont I., Vasquez-Lazo J., Subirana I., Schroder H. (2010). Obesity is an independent risk factor for heart failure: Zona Franca Cohort study. Clin. Cardiol..

[14] Zahr F., Genovese E., Mathier M., Shullo M., Lockard K., Zomak R., McNamara D., Toyoda Y., Kormos R.L., Teuteberg J.J. (2011). Obese patients and mechanical circulatory support: Weight loss, adverse events, and outcomes. Ann. Thorac. Surg..

[15] Zhigalov K., Szczechowicz M., Mashhour A., Mkalaluh S., Isaev M., Kadyraliev B., Easo J., Ennker J., Eichstaedt H., Weymann A. (2019). Impact of previous sternotomy on outcome after left ventricular assist device implantation. Thorac. Cardiovasc. Surg..

[16] Zhigalov K., Szczechowicz M., Mashhour A., Kadyraliev B.K., Mkalaluh S., Easo J., Ennker J., Eichstaedt H.C., Weymann A. (2019). Left ventricular assist device implantation with concomitant tricuspid valve repair: Is there really a benefit?. J. Thorac. Dis..

[17] Harrington G., Russo P., Spelman D., Borrell S., Watson K., Barr W., Martin R., Edmonds D., Cocks J., Greenbough J. (2004). Surgical-site infection rates and risk factor analysis in coronary artery bypass graft surgery. Infect. Control Hosp. Epidemiol..

[18] Yap C.H., Mohajeri M., Yii M. (2007). Obesity and early complications after cardiac surgery. Med J. Aust..

[19] Omalu B.I., Ives D.G., Buhari A.M., Lindner J.L., Schauer P.R., Wecht C.H., Kuller L.H. (2007). Death rates and causes of death after bariatric surgery for Pennsylvania residents, 1995 to 2004. Arch. Surg..

[20] Dhesi P., Simsir S.A., Daneshvar D., Rafique A., Phan A., Schwarz E.R. (2011). Left ventricular assist device as’ bridge to weight loss’ prior to transplantation in obese patients with advanced heart failure. Ann. Transplant..

[21] Clerkin K.J., Naka Y., Mancini D.M., Colombo P.C., Topkara V.K. (2016). The impact of obesity on patients bridged to transplantation with continuous-flow left ventricular assist devices. JACC Heart Fail..

[22] Coyle L.A., Ising M.S., Gallagher C., Bhat G., Kurien S., Sobieski M.A., Slaughter M.S. (2010). Destination therapy: One-year outcomes in patients with a body mass index greater than 30. Artif. Organs.

[23] Butler J., Howser R., Portner P.M., Pierson R.N. (2005). Body mass index and outcomes after left ventricular assist device placement. Ann. Thorac. Surg..

[24] Brewer R.J., Lanfear D.E., Sai-Sudhakar C.B., Sundareswaran K.S., Ravi Y., Farrar D.J., Slaughter M.S. (2012). Extremes of body mass index do not impact mid-term survival after continuous-flow left ventricular assist device implantation. J. Heart Lung Transplant..

[25] Mohamedali B., Yost G., Bhat G. (2015). Obesity as a risk factor for consideration for left ventricular assist devices. J. Card. Fail..

[26] Yost G., Coyle L., Gallagher C., Graney N., Siemeck R., Tatooles A., Pappas P., Bhat G. (2017). The impact of extreme obesity on outcomes after left ventricular assist device implantation. J. Thorac. Dis..

[27] Musci M., Loforte A., Potapov E.V., Krabatsch T., Weng Y., Pasic M., Hetzer R. (2008). Body mass index and outcome after ventricular assist device placement. Ann. Thorac. Surg..

[28] Birati E.Y., Jessup M. (2015). Left ventricular assist devices in the management of heart failure. Card. Fail. Rev..

[29] Samad F., Ruf W. (2013). Inflammation, obesity, and thrombosis. Blood.

[30] Darvall K.A., Sam R.C., Silverman S.H., Bradbury A.W., Adam D.J. (2007). Obesity and thrombosis. Eur. J. Vasc. Endovasc. Surg..

[31] Han J.J., Sooppan R., Johnson A.P., Chen C.W., Gaffey A.C., Phillips E.C., Howard J., Rame J.E., Acker M.A., Atluri P. (2017). Higher body mass index increases risk of HeartMate II pump thrombosis but does not adversely affect long-term survival. Circ. J..

[32] Bandaru H., Afzal M.R., Maud A., Kassar D., Khatri R., Rodriquez G., Piriyawat P., Cruz-Flores S., Vellipuram A. (2019). Neurological Complications of Left Ventricular Assist Device. Neurology.

